# Breast cancer risk in premalignant lesions: osteopontin splice variants indicate prognosis

**DOI:** 10.1038/s41416-018-0228-1

**Published:** 2018-10-24

**Authors:** Kinga Walaszek, Elyse E. Lower, Piotr Ziolkowski, Georg F. Weber

**Affiliations:** 1 Department of Pathology, Wroclaw Medical University, Wroclaw, Poland; 2 College of Medicine, University of Cincinnati Academic Health Center, Cincinnati, OH USA; 3 College of Pharmacy, University of Cincinnati Academic Health Center, Cincinnati, OH USA

## Abstract

**Background:**

Premalignant breast lesions pose variable risks for transformation, raising the question who should receive treatment to counteract the potential progression to breast cancer. Because the secreted metastasis mediator Osteopontin (OPN) is a marker for breast cancer aggressiveness, its presence in these lesions may reflect progression risk.

**Methods:**

By immunohistochemistry, we analyse the association of Osteopontin variant expression in healthy breasts, hyperplasias, papillomas, and carcinomas in situ from 434 women to assess a) staining for OPN exon 4 (present in OPN-a and OPN-b) or OPN-c in low-risk to high-risk lesions b) correlations between staining and progression (DCIS with invasion, invasive cancer) or survival.

**Results:**

The markers correlate with risk, and they are prognostic for ensuing invasive disease and survival. About 10% of OPN-c pathology score 0–1 (intensity), vs. 40% of score 3 experience cancer over 5 years. More than 90% of women, who progress, had pathology scores of 2–3 for OPN-c intensity at the time of initial diagnosis. When combining OPN-c and OPN exon 4 staining, all of the low intensity patients are alive after 5 years, whereas women in the high category have a close to 30% chance to die within 5 years. Of patients who succumb, close to 80% had a high combined score at the time of initial diagnosis.

**Conclusion:**

The combined information of OPN splice variant immunohistochemistry can provide a foundation for very reliable prognostication and has the potential to aid decision making in the treatment of early breast lesions.

## Introduction

The progress achieved in imaging and detection over recent years has generated a relatively new dilemma in breast disease: Which patients with premalignant lesions should receive treatment to prevent the future development of breast cancer? While such changes are present in about 5% of disease-free women, their clinical significance is uncertain as not all cases progress.^1,2^ Women with preinvasive disease have three options, observation, chemoprevention (mostly with selective estrogen receptor modulators or aromatase inhibitors), or surgery (lumpectomy or mastectomy). It is difficult for the individual patient to make that choice because there are no predictors for her specific progression risk. A molecular diagnostic that informs the patient whether she is at high or low risk for developing breast cancer can facilitate the decision on follow-up treatment. Distinguishing high-risk patients from low-risk patients will improve the prognosis of the former group (through early decisive intervention) and spare unnecessary treatment for the latter group (through watchful waiting).

Normal histology or usual ductal hyperplasia put a patient at low risk for developing breast cancer. Early stages of breast transformation develop from hyperplasia to atypia (flat epithelial atypia (FEA), atypical ductal hyperplasia (ADH)^3,4^), papillomatosis or lobular carcinoma in situ (LCIS) with moderate risk for transformation. Non-invasive, but potentially precancerous lesions are called ductal carcinoma in situ (DCIS). DCIS is characterised by the proliferation of transformed epithelial cells within ducts, which are surrounded by an intact basement membrane. There is a 30–50% risk that DCIS (stage 0), if not treated, will progress to locally invasive breast cancer and then to metastatic breast carcinoma (stage III). The acquisition of invasiveness is a critical step in these early breast carcinomas. It is associated with the aberrant expression and splicing of specific tumour progression genes that allow the cells to penetrate the basement membrane.^5^ While there is a substantial need in breast cancer progression to identify biomarkers for the sequence: hyperplasia → atypia/papilloma → DCIS → DCIS with microinvasion → invasive ductal cancer (IDC), or alternatively from atypia via LCIS to invasive lobular cancer (ILC), current breast histopathology does not allow the reliable diagnosis of this invasive potential.

Biomarkers are important for guiding the diagnosis and management of growths in the breast. Two broad groups of biomarkers comprise prognostic markers and predictive markers. Prognostic markers allow forecasts regarding the natural course of the disease. They differentiate between patients likely to have a good vs. a poor outcome. By contrast, predictive markers provide upfront information regarding how likely a patient is to benefit from a specific treatment, and hence may guide the choice from available therapies. Two of the most critical questions in breast cancer, for which there is a paucity of suitable biomarkers, comprise the prediction of treatment responses and the prognostication which premalignant breast lesions will form cancer. The cytokine Osteopontin (OPN, Spp1) has been extensively studied as a metastasis gene. It constitutes the most abundantly secreted phospho-protein in breast and other cancers and supports invasive behaviour. As such, it is a biomarker for breast cancer aggressiveness and for breast cancer prognosis (the abundance of Osteopontin correlates negatively with survival). In older studies, pan-Osteopontin (total Osteopontin, typically covering all variant forms) was measured.^6,7^ However, the gene product is subject to alternative splicing selectively in cancer, which deletes exon 4 (to generate Osteopontin-c) or exon 5 (to generate Osteopontin-b) from the unspliced form (called Osteopontin-a). The variants have distinct pathophysiological functions in cancer progression and convey distinct information on the disease. We have previously investigated the predictive capabilities of splice variants for treatment responses^8^ and their prognostic potential for cancerous lesions.^9,10^ A prior meta-analysis confirmed pan-Osteopontin to be correlated with premalignant progression in breast and other transformations.^6^ Here we analyse the prognostic value of OPN splice variants in mammary tissue at the premalignant stage. The variant forms are distinguishable by antibodies to exon 4, recognising OPN-a and OPN-b, or to the splice junction of OPN-c respectively.

## Materials and methods

### Patients

This study investigated biopsies from a total of 434 women with premalignant breast lesions (Table 1); comprising 343 patients from Wroclaw, Poland and 91 patients from Cincinnati, USA. The diagnoses range from healthy breasts or usual ductal hyperplasia via atypia/atypical ductal hyperplasia or papillary breast lesions or lobular carcinoma in situ to ductal carcinoma in situ. The papillary breast lesions encompass a spectrum of masses, which present as fronds attached to the inner mammary duct wall by a fibrovascular core with both epithelial and myoepithelial cells; although not malignant, papillary disease is associated with an increased risk of invasive breast cancer. In LCIS, abnormal cells start growing in the lobules, the milk-producing glands at the end of breast ducts. In DCIS, the presence of abnormal cells inside milk ducts poses an elevated risk for breast cancer. All DCIS patients had a resection, 12 were treated with tamoxifen alone, 39 with radiation alone, and 41 with tamoxifen plus radiation therapy. The patients in Poland were followed up to 5 years, the patients in the US initially presented 2005–2011. The Cincinnati patients were not sequential specimens. Because the incidence of invasion after DCIS or atypia is very low, they were selected in two groups, those who subsequently developed invasive cancer and those who did not. The lead investigator and biostatisticians were blinded to this selection. The study was approved by the ethics committees at Wroclaw Medical University, Poland and the University of Cincinnati, USA.

**Table 1:** Patient characteristics

Risk group	Diagnosis	Acronym	*N*	Survival			Progression			Treatment	
				Dead	Alive	No data	Pro-gression	Non-progression	No data	Tam-oxifen	Radiation
Low risk	Sine neoplasmate (without lesions)	SN	54	0	12	42	0	12	42	0	0
	Usual ductal hyperplasia	UDH	60	0	20	40	0	20	40	0	0
Intermediate risk	Atypical ductal hyperplasia	ADH	74	0	55	19	6	53	15	0	0
	Papillomatosis intraductalis/papilloma	PI	19	0	6	13	0	6	13	0	0
	Lobular carcinoma in situ	LCIS	20	2	13	5	5	13	2	0	0
Elevated risk	Ductal carcinoma in situ	DCIS	198	31	123	44	57	104	37	53	80
(risk level controversial)	Radial scar (sclerosing duct hyperplasia)	RAD SCAR	9	0	7	2	1	6	2	0	0
	(all groups)		434								

Shown are the risk groups and diagnoses (full names and acronyms). Follow-up information on progression or death was available for a fraction of the patients and is depicted by survival (dead, alive, no data), progression (progression, non-progression, no data) and treatment (tamoxifen, radiation). *N* = number of patients; the bottom row shows the total number of patients studied. The patients in Poland presented over the past 5 years, the patients in the United States initially presented 2005–2011. Hence, where follow-up information was available, it ranged from 1 to 11 years (Figs. 3, 4, Supplement 2 analyse the canonical 5-year follow-up)

### Immunohistochemistry

For each antibody, a formalin-fixed and paraffin-embedded biopsy specimen from premalignant lesions were cut on a microtome in 5 μm slices. The antibodies used in this study, after blocking in 2% donkey serum, were anti-hOPN-c IgY (Georg F. Weber, distributed by Gallus Immunotech), and LF161 (Larry Fisher). The IgY antibody recognises the Osteopontin-c splice junction and detects the molecule in immunohistochemistry. It was diluted 1:500 to 1:700. The polyclonal rabbit antibody LF161 for staining selectively exon 4 (present in Osteopontin-a and -b) was used at 1:1000. The antibodies and their use in immunohistochemistry have been thoroughly validated.^9,10,11^ For each antibody, the tissues were scored according to intensity (maximum intensity of the sample 0, 1, 2, or 3) and percent positivity (0, 1, 2, or 3), separately for nuclei and cytoplasm. In addition to analysing the indicators in their original scale, we dichotomised the immunohistochemical biomarkers into low (0–1) or high (2–3). We have previously found this method to strengthen the power of the analysis.^8^ All microscopic slides were independently evaluated by two pathologists, and in the rare cases of discrepant initial scores, a final score was agreed on after discussion.

### Statistics

Statistical analyses were conducted using MedCalc version 14.8.1. The pathology scores assess staining intensity and percent positivity. The predictors were each categorical or dichotomised (pathology scores 0 and 1 = low vs. 2 and 3 = high). A second analysis included the risk group. For evaluating differences in biomarkers among the risk groups (obtained from pathology scores and the premalignant diagnoses) a χ^2^ test was applied. The primary methods for addressing survival time (duration) and prognosis (ensuing invasive disease or death) was Kaplan Meier for univariate analysis. A multivariate analysis of those factors with a p-value of less than 0.05 were then applied to a Cox proportional hazard model. The hazard ratio (HR) measures the hazard between two individuals, whose value of the independent variable differ by one unit (if continuous) or moving from one class to another class (for categorical variable).

### Logistic regression

For biomarker development, we devised ROC curves using all parameters (intensity and percent positivity of the immunohistochemistry stains plus risk groups). To model outcome (survival or progression). We used the formula

$$\begin{array}{ccccc}\\ & \pi \left( {X_1,X_2,X_3,X_4,X_5} \right) & = \displaystyle \frac{{e^{\beta _0 + \beta _1X_1 + \beta _2X_2 + \beta _3X_3 + \beta _4X_4 + \beta _5X_5}}}{{1 + e^{\beta _0 + \beta _1X_1 + \beta _2X_2 + \beta _3X_3 + \beta _4X_4 + \beta _5X_5}}}\\ \end{array}$$

with *X* indicating the parameters measured (4 pathology scores as categorical variables and risk as a dichotomised variable) and β being the coefficient for the regression (calculated in *R*, a language and environment for statistical computing and graphics). The log odds are a linear function of the covariates.

## Results

### Patient characteristics

Of 434 patients, 54 women had healthy breast tissue (sine neoplasmate) and 60 had usual ductal hyperplasia. These two groups are considered to be at low risk for progression. Atypia/ADH (74 cases), papilloma/papillomatosis intraductalis (19 cases) and LCIS (20 cases) comprise an intermediate progression risk (risk of subsequent invasive cancer increases from 1.5 to 2.0% for proliferative lesions without atypia, to 3.5–5.0% for hyperplastic lesions with atypia^12^). The 198 patients with DCIS (ductal carcinoma in situ) have an elevated risk to develop breast cancer. The risk level of radial scar is not fully characterised; 9 patients with this diagnosis were included in the overall evaluation without assignment to risk groups (see Table 1). Follow-up information was available for a fraction of the subjects as indicated below.

### Immunohistochemistry

**Fig. 1:**
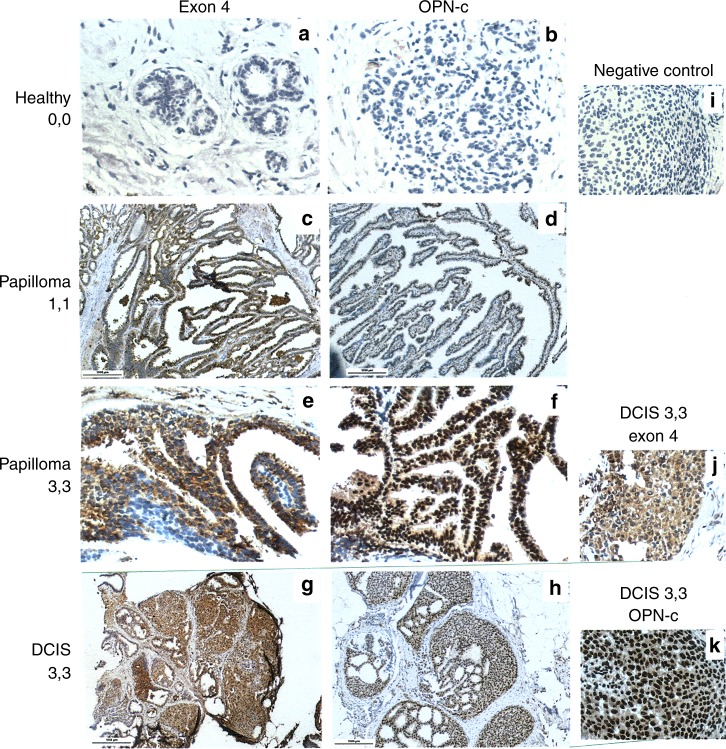
Immunohistochemistry of the patient samples. All specimens were developed using the LSAB (labelled Streptavidin-Biotin) method. The original magnification was ×100 (please note the scale bars). The left column (**a**, **c**, **e**, **g**) shows staining for OPN exon 4, the middle column (**b**, **d**, **e**, **h**) shows staining for OPN-c. The right column (**i**, **j**, **k**) contains additional information. **a**, **b** Healthy breast tissue with a lack of staining for OPN exon 4 (**a**) and a lack of staining for OPN-c (**b**) (intensity of staining, percent of stained cells = 0, 0). **c**, **d** Intraductal papillomatosis with weakly positive cytoplasmic staining for OPN exon 4 (**c**) and weakly positive nuclear staining for OPN-c (**d**) (1, 1). **e**, **f** Intraductal papillomatosis with strongly positive cytoplasmic staining for OPN exon 4 (**e**) and nuclear staining for OPN-c (**f**) (3, 3). **g**, **h** Ductal carcinoma in situ (DCIS) with strongly positive cytoplasmic staining for OPN exon 4 (**g**, larger magnification in **j**) and strongly positive nuclear staining for OPN-c (**h**, larger magnification in **k**) (3, 3). **i** DCIS negative control (no staining; first antibody was omitted). **j**, **k** larger magnification of a strongly positive DCIS confirms the predominantly cytoplasmic staining for OPN exon 4 (**j**) and the predominantly nuclear staining for OPN-c (**k**) (the green lines connect low and high magnification for the same type of staining)

The anti-Osteopontin exon-4 antibody, which recognises Osteopontin-a and -b, stained selectively the cytoplasm. Lesions displayed Osteopontin-c predominantly in their nuclei (lesion-free breasts had no staining) (Fig. 1). The markers (OPN-c nuclear intensity, OPN-c nuclear percent positivity, exon 4 cytoplasmic intensity, exon 4 cytoplasmic percent positivity) showed increases in average pathology scores with higher transformation risk (from low via intermediate to high). OPN-c was more stringently associated with the elevated risk groups than exon 4, reaching significant p-values for staining intensity as well as for percent positivity in all comparisons. Further, for each subgroup comparison, OPN-c staining intensity and percent positivity, but not OPN exon 4 staining, reached significant levels of difference between diagnostic entities (Table 2).

**Table 2:** Pathology scores in distinct subgroups correlate with risk

			OPNa/b		OPNc	
			Intensity	Percent	Intensity	Percent
Low risk	Intermediate risk	χ^2^	9.635	5.065	68.385	57.794
		*P*	0.022	0.167	<0.0001	<0.0001
Low risk	Elevated risk	χ^2^	27.600	27.703	129.889	134.821
		*P*	<0.0001	<0.0001	<0.0001	<0.0001
Intermediate risk	Elevated risk	χ^2^	18.753	16.021	14.868	15.635
		*P*	0.000	0.001	0.002	0.001
SN	ADH	χ^2^	8.313	12.704	72.489	62.720
		*P*	0.040	0.005	<0.0001	<0.0001
UDH	ADH	χ^2^	8.151	1.752	19.261	10.382
		*P*	0.043	0.625	0.000	0.016
SN	DCIS	χ^2^	48.796	49.294	176.546	176.087
		*P*	<0.0001	<0.0001	<0.0001	<0.0001
UDH	DCIS	χ^2^	5.018	4.977	49.278	54.982
		*P*	0.171	0.174	<0.0001	<0.0001
ADH	DCIS	χ^2^	15.007	14.304	15.401	16.015
		*P*	0.002	0.003	0.002	0.001

χ^2^ test for differences in pathology scores (staining intensity followed by percent positivity) among various premalignant diagnoses. *P* = p-value (underlined if lower than 0.05). The upper portion of the Table shows the evaluation of the main risk groups; low risk comprises SN (sine neoplasmate) and UDH (usual ductal hyperplasia); intermediate risk entails atypia/ADH (atypical ductal hyperplasia), papilloma/papillomatosis, LCIS (lobular carcinoma in situ); elevated risk is DCIS (ductal carcinoma in situ). The lower section compares pairwise the diagnostic subgroups with the largest patient numbers. OPNa/b denotes staining for exon 4, OPNc denotes staining for the splice junction of OPN-c

### Prognosis

**Fig. 2:**
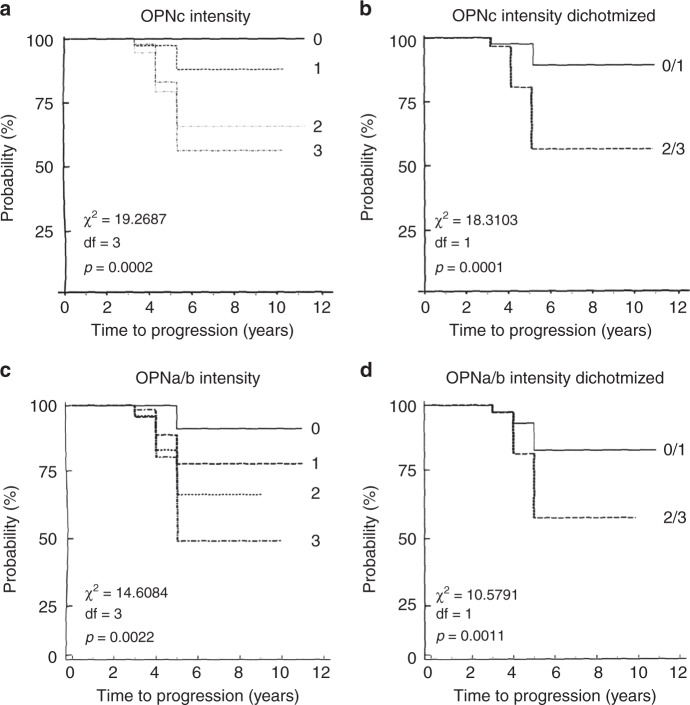
OPN splice variants are indicators for prognosis. Kaplan–Meier curves for the risk of progression over time. The *x*-axis shows the time of follow-up in years, the *y*-axis displays the probability of remaining recurrence-free cases in percent of the total number. The measured variables in **a**, **c** are categorical. The variables in **b**, **d** are dichotomised. The χ^2^ statistic is inserted into the lower left corner of the graphs, df degrees of freedom. **a** OPN-c intensity. **b** OPN-c intensity dichotomised. **c** OPN exon 4 intensity. **d** OPN exon 4 intensity dichotomised

Follow-up information had 214 patients with non-recurrence over various observation periods (111 were free of relapse for at least 5 years following the initial diagnosis) and 55 patients (20%) experiencing breast cancer over 3–5 years (48 patients had insufficient follow-up duration or died from other causes and were excluded). The data identified OPN-c intensity scores 2–3 as stronger predictors for progression than intensity scores 0–1 for all types of lesions analysed (Supplementary Figure 1). For OPN exon 4, the probability of progression increased with score, and moderate gain was achieved by dichotomising (Fig. 2). The dichotomised scores were used for biomarker development (see below). Multi-variate analysis confirmed that the two biomarkers OPN-c and OPN exon 4 are prognostic for ensuing invasive disease, whereas the risk group did not add significantly to the prognostication (consistent with reports that OPN-c is a progression marker for all types of breast cancer^10^). Among the risk groups, expectedly, DCIS was associated with the highest probability of developing breast cancer compared to PI, ADH, and LCIS (Supplementary Figure 2). A Cox proportional hazards regression model was applied for the variables under consideration. OPN-c intensity had a *p*-value of 0.0022 and a hazard ratio of 1.8181 (95% confidence limits 1.2427–2.6597). OPN-a/b intensity had a *p*-value of 0.0220 and a hazard ratio of 1.4456 (95% confidence limits 1.0564–1.9783). By contrast, the values for risk were *p*-value 0.7185, hazard ratio 0.9472 (95% confidence limits 0.7064–1.2702). This suggests that the OPN variant forms are biomarkers for progression hazard, also for lesions that are conventionally categorised as low risk. The markers may be of particular benefit in assessing the need for treatment in non-DCIS premalignant lesions.

### Biomarker properties

**Fig. 3:**
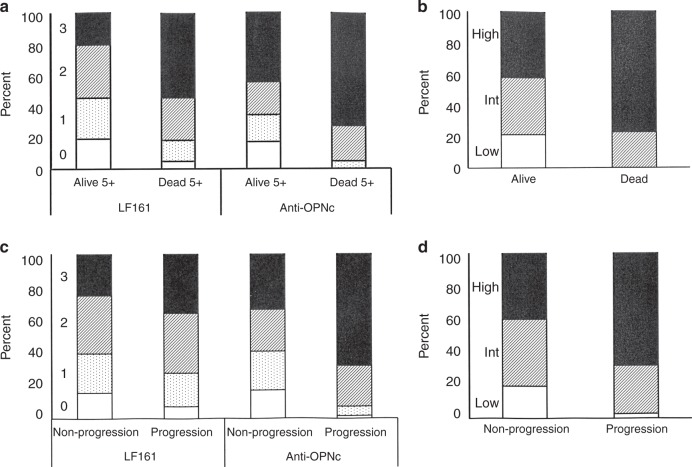
Pathology scores prognosticate outcome subgroups. **a**, **c** Shown are the distributions of pathology scores for marker staining intensity (0–3 as indicated to the left of the bars, with 0 = unfilled, 1 = dotted, 2 = hatched, 3 = filled) for 139 patients who do or do not succumb to cancer (**a**) and 140 patients who do or do not experience progression (**c**) over a time frame of 5 years (5 + indicates that some patients remained free of adverse outcome over more than 5 years, shorter follow-ups are disregarded). The left two bars reflect LF161 staining (OPN exon 4), whereas the right two bars indicate anti-OPN-c staining. The y-axis shows the number of patients as percent of the total in each group. **b**, **d** To utilise the information of both markers and to gain discrimination, the pathology scores for OPN-c and OPN exon 4 marker intensities were combined and their distributions visualised on stacked bar graphs (low = pathology scores for both markers 0–1, intermediate = one marker 0–1 the other 2–3, high = both markers 2–3, as indicated to the left of the bars; low = unfilled, intermediate = hatched, high = filled) for patients who do or do not succumb to cancer (**b**) and patients who do or do not experience ensuing invasive disease (**d**) over a time frame of 5 years. The *y*-axis shows the number of patients as percent of the total in each group

We evaluated patients who died from breast cancer within 5 years in comparison to those who were alive for at least 5 years following the initial diagnosis. Analysis for the association of outcome with the markers under investigation (OPN-c, OPN exon 4) reflected them as prognostic. The pathology scores were higher for OPN exon 4 as well as for OPN-c in patients who succumbed to breast cancer compared to those who over at least 5 years did not. When combining OPN-c and OPN exon 4 staining intensity on a scale of low (pathology scores for both markers 0–1), intermediate (one marker 0–1 the other 2–3) and high (both markers 2–3), the prognostic accuracy improved such that all of the low patients were alive after 5 years, whereas women in the high category had a 30% chance to die within 5 years (with almost 20% of the survivors among them having experienced documented invasive disease). Close to 80% of patients who succumbed had a high score at the time of initial diagnosis (Fig. 3a, b). In the intermediate group, a high score for OPN-c was more unfavourable (ratio alive:dead = 6.5:1) than a high score for exon 4 (ratio alive:dead = 15:1).

We analysed patients who incurred breast cancer within 5 years in comparison to those who were free of relapse for at least 5 years following the initial diagnosis. More than 90% of women, who experienced breast cancer had had pathology scores of 2–3 for OPN-c intensity at the time of initial diagnosis. About 2.5% of women free of OPN-c (intensity pathology score 0), and 7.5% of OPN-c pathology score 1 progress over 5 years. This risk increases to 24% at pathology score 2 and 40% at pathology score 3. However, OPN exon 4 was less informative than OPN-c (13% at intensity score 0, 21% at score 1, 25% at score 2, 31% at score 3), so that combining the two markers yielded modest improvement over OPN-c intensity alone (Fig. 3c, d).

**Fig. 4:**
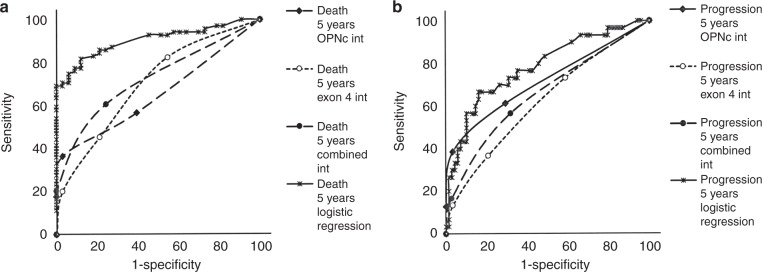
ROC curves validate the OPN splice variants as markers for progression and survival. Shown are graphs for survival (**a**) and progression (**b**). All indicators are above the diagonal. Evaluated are the pathology scores for OPN-c staining intensity alone, OPN exon 4 staining intensity alone, both markers combined, and a logistic regression analysis including all of the variables under study. The non-smooth curve-fit for the combined analysis reflects the iterations of the logistic regression as calculated in R. int = staining intensity, combined = sum of pathology scores for OPN-c and OPN exon 4 staining

According to ROC curves, a logistic regression algorithm that applies the pathology scores as categorical variables and the dichotomised risk group (low or medium vs. high) achieves better sensitivity and specificity for the prognostication of death from breast cancer (Fig. 4a), as well as for the prognostication of cancer development (Fig. 4b) than any of the individual pathology scores alone. The combined information derived from OPN-c staining, OPN exon 4 staining, and diagnosis can provide a foundation for very reliable prognostication.

### DCIS treatment

All DCIS cases underwent surgical resection (16 patients had mastectomies). A fraction of them was further treated with tamoxifen (12), radiation (39) or both (41). While the sizes of the subgroups preclude conclusive assessments due to lack of statistical power, the trends suggest that the prognostic value of OPN-c/exon 4 is insignificantly affected by ensuing treatment, and that within each treatment group (either tamoxifen plus radiation or tamoxifen alone) the biomarker may be able to distinguish high vs. low risk for invasive disease (Supplement Fig. 3).

## Discussion

In this study, we have identified OPN splice variant -c as a prognostic indicator for ensuing invasive disease and survival following premalignant breast lesions. OPN exon 4 and the diagnosis of the lesion are contributing markers. The observation is consistent with existing knowledge regarding the biological effects of the splice variants. Although the spliced OPN forms are always expressed together with the full-length form OPN-a, their ratios vary (the rate of RNA splicing is different from, and functionally independent of the rate of transcription). While OPN-a and OPN-c may synergise in tumour progression,^13^ OPN-c is more potent in promoting aggressive behaviour.^14^ OPN splice variants have been found to be of value for breast cancer diagnosis/prognosis/prediction (Supplementary Table 1). Adding measurements of OPN-c and OPN exon 4 to existing diagnostic workups of precancerous lesions holds promise for assessing invasive potential and for prognosticating cancer risk, which existing markers cannot do.

Available diagnostic techniques in breast cancer prevention involve a biopsy, where samples of tissue are taken to confirm or eliminate the presence of transformed cells by histopathological examination. While this procedure is a standard at present, it lacks indicators for the detection of invasive potential. Most biopsies are obtained with a core needle, and sampling or interpretation error may understate the disease identified.^15^ The early stages of breast transformation (atypias) are difficult to differentiate from benign growths (hyperplasias) on one end of the spectrum and invasive carcinomas in situ (CIS) on the other end. Furthermore, the inspection of tumour margins to assess invasiveness is unreliable and it requires step sections through the entire biopsy material. Microinvasion is typically identified at surgical biopsy, as core needle tissue cannot enable this distinction. Hence, there is an intense need for reliable biomarkers. We have analysed biopsies, and there is a possibility that an invasive component was present, but cryptic to histologic analysis at that time (in the patient records, post-surgical corroboration of a non-cancer/DCIS diagnosis was available only for a fraction of the women). The independent variable OPN-c/exon 4 enables the assessment of risk regardless of the type of premalignant lesion present. Because its staining intensity is indicative of invasive potential, it allows histopathologic evaluation even when the margins cannot be inspected. The prognostic biomarker is not dependent on the two most common constraints, histologic type and margins of the lesion. Its value lies in being able to examine invasive potential, regardless whether an invasive component has been missed in the biopsy or invasion has not yet occurred.

While research has recognised a large number of biomolecules to be deregulated or defective in breast cancer, relatively few of them are commonly used in histological diagnosis. Specifically, markers that predict invasiveness have not been firmly established, and they have been sorely absent from utilisation in premalignant lesions. Among the accepted molecular indicators, the U.S. FDA-approved and ASCO-recommended tumour markers CA15.3, CA27.29, and CEA are useful only for monitoring the therapy of advanced breast cancer or its relapse. These serum markers still lack the adequate sensitivity (below 25%) and specificity (below 70%) to be applicable in diagnosing early stage breast carcinoma in a large population.^16,17^ Estrogen receptor (ER) and progesterone receptor (PR) facilitate decisions on therapy, but are weak prognostic measures.^10,16,18^ HER2 over-expression is associated with poor prognosis, but HER2-based mechanisms underlie only about one third of breast cancers.

Genetic signatures have been used to assess risk in more advanced lesions. The oncotype Dx relapse score involves 21 genes. It has prognostic utility for relapse and survival in tamoxifen-treated, node-negative, ERα-positive cases. It permits the identification of a subgroup with sufficiently low residual risk to safely omit chemotherapy. It may also be informative in low nodal positivity, identifying patients who can benefit from anthracycline-based chemotherapy. The MammaPrint signature comprises 70 genes that identify risk for relapsing within 5 years. It is applicable to node-negative, ER-negative patients. High risk women benefit significantly from the addition of chemotherapy to endocrine treatment. Although subject to active research,^19^ no such tests have been developed for precancerous lesions.

While the proliferation marker Ki-67 has shown some prognostic potential for risk assessment in premalignant breast disease,^20^ the value of indicators can be improved by using the presence of molecules that are essential for invasion through tissue barriers, which constitutes a critical transformation step. Published reports include the polycomb group transcriptional repressor EZH2, which is elevated in invasive breast carcinoma compared with normal breast epithelia.^21^ VEGF correlates with uPA in the node-positive population, and patients with high VEGF levels display poor outcome, with an increased risk for the node-positive subset.^22^ Neither of these potential molecular indicators has been developed for routine diagnostic use. Further research will indicate whether combining OPN splice variants with other candidate markers can further improve their prognostic potential in premalignant breast disease.

## Electronic supplementary material

Supplement
