# Supplementary material for: Breast cancer risk in premalignant lesions: osteopontin splice variants indicate prognosis
Source: Br J Cancer. 2018 Oct 24;119(10):1259–66. doi: 10.1038/s41416-018-0228-1 (PMC6251032; doi:10.1038/s41416-018-0228-1)
Supplement: Supplementary file 1 — Supplement [file 41416_2018_228_MOESM1_ESM.docx]

**Supplement Table 1:** **OPN splice variants in breast cancer.** Variant forms of OPN have been measured on the protein and RNA levels, in tumor tissue and in blood. These markers are associated with various disease characteristics in breast cancer. The patient numbers were 22 breast cancers/20 controls (RNA)^10^, 56 breast cancers/69 controls (protein)^10^, 671 breast cancers^9^, 170 breast cancers/30 controls^23^, 415 breast cancers^24^, 127 breast cancers/33 controls^25,26^, 119 breast cancers^8^, 67 breast cancers/74 controls^27^.

**Supplement Figure 1: Survival, reflected in Kaplan-Meier Curves, is prognosticated by OPN-c staining.** Survival curves in relation to OPN-c staining intensity for all patients with follow-up information (left panel; N = 42, 122), DCIS patients (middle panel; N = 19, 82), and patients with premalignant lesions other than DCIS (right panel; N = 23, 40). The change in Y-axis scale is intended to highlight the difference between OPN-c low (intensity score 0-1) and OPN-c high (intensity score 2-3), even though the risk for recurrence differs among the subgroups of patients.

**Supplement Figure 2:** **Multivariate analysis for the combination of prognostic indicators.** **A)** Evaluated were the pathology scores for OPN exon 4 and OPN-c together with risk group for prognosticating recurrence. The graph represents a Kaplan-Meier curve for the risk of recurrence over time. The x-axis shows the time of follow-up in years, the y-axis displays the probability of remaining recurrence-free cases. **B)** Kaplan-Meier curves for the risk of recurrence over time by diagnosis. The x-axis shows the time of follow-up in years, the y-axis displays the probability of remaining progression-free cases. The measured variables are categorical. The χ^2^ statistic is inserted into the lower left corner of the graph, df = degrees of freedom. In both graphs, the scale of the y-axis is stretched to maximize resolution.

**Supplement Figure 3: DCIS outcome under treatment.** Distributions of combined pathology scores for OPN-c and OPN exon 4 marker intensities (open box = low = pathology scores for both markers 0-1, hatched box = intermediate = one marker 0-1 the other 2-3, filled box = high = both markers 2-3) for patients who do or do not experience invasive disease over a time frame of 5 years. **A)** The distribution of combined pathology scores differs only moderately among treatment groups (tamoxifen+radiation n=41, radiation n=39, tamoxifen n=12). **B)** The distribution of combined pathology scores differs only moderately between tamoxifen-treated and not tamoxifen-treated patients (tamoxifen n=53, not treated n=62). **C and D)** Among treated patients, the combined pathology score may predict risk, but very low numbers require confirmation in a larger patient population. **C)** Tamoxifen and/or radiation (non-progression n=59, progression n=3). **D)** Tamoxifen (non-progression n=4, progression n=3).
